# Assessment of subjective well-being of healthcare workers in response to heat and personal protective equipment under controlled conditions using a standardized protocol

**DOI:** 10.1186/s12995-024-00418-5

**Published:** 2024-05-15

**Authors:** Caroline Quartucci, Razan Wibowo, Viet Do, Stephan Bose-O`Reilly, Dennis Nowak, Veronika Weilnhammer, Tobias Weinmann, Stefan Rakete

**Affiliations:** 1 Institute and Clinic for Occupational, Social and Environmental Medicine, LMU University Hospital, LMU Munich, Munich, Germany; 2 Institute for Occupational Health and Product Safety, Bavarian Health and Food Safety Authority, Environmental Health, Munich, Germany; 3 Department of Public Health, Medical Informatics and Technology, Health Services Research and Health Technology Assessment, UMIT - University for Health Sciences, Hall in Tirol, Austria

**Keywords:** Heat stress; climate change; occupational safety, Health care workers, Personal protective equipment

## Abstract

**Background:**

Due to climate change, the increasing frequency of hot summer days and heat waves can result in occupational heat strain, especially in non-air-conditioned workplaces. Healthcare workers (HCW) engaged in patient care are particularly affected, as they are additionally exposed to physical stress. The use of personal protective equipment (PPE) can aggravate heat strain in HCW. This study aimed to examine the subjective well-being of HCW when exposed to heat and PPE under controlled conditions.

**Methods:**

This study was designed as a randomized crossover trial. Participants performed standardized healthcare tasks in a climatic chamber for approximately 3.5 h at different indoor temperatures (22 °C and 27 °C) and varied working conditions (with or without PPE). The effects on participants’ subjective well-being, encompassing thermal, physiological and psychological stress were assessed using a customized questionnaire.

**Results:**

Heat had a greater effect than PPE on thermal, physical and psychological stress. Conversely, PPE had a greater effect on physical demand and effort. For the majority of outcomes, combined exposure to heat and PPE resulted in the highest perceived discomfort. Furthermore, the participants reported increased sweating and other discomforts when working at elevated temperatures or with PPE.

**Conclusions:**

In this study, heat and PPE, but particularly the combination of both factors, were identified as unfavorable working environments. Although the trials were conducted in a controlled environment, the outcomes provide valuable information about the effect of heat and PPE on HCW in a real-life setting. Furthermore, the design used in this study can be beneficial in evaluating the effect of mitigation strategies.

**Supplementary Information:**

The online version contains supplementary material available at 10.1186/s12995-024-00418-5.

## Background

Climate change has been identified as one of the most pressing challenges of the twenty-first century [1]. Moreover, rising global temperatures are closely linked to adverse health effects including dehydration [2], cardiovascular disease [3] and kidney disease [4, 5]. Especially elderly people and people with pre-existing medical conditions as well as workers with increased exposure to hot ambient environments are particularly vulnerable to adverse health effects. It has been demonstrated that elevated temperatures at workplaces are an emerging occupational hazard that can reduce work capacity and motor-cognitive performance, adversely affecting productivity [4, 6, 7]. In terms of healthcare workers (HCW), hot periods in combination with the use of personal protective equipment (PPE) in non-air-conditioned healthcare facilities bears the risk of developing heat related health problems [8, 9]. PPE (e.g., N95 mask, face shield, gown, gloves) provides protection against biological hazards and is required to care for (potentially) infectious patients. However, PPE reduces the dissipation of body heat and, thus, potentially aggravates heat stress [10].

Numerous studies have highlighted the impact of working with PPE on various aspects of occupational health. Studies by Dorman and Havenith (2009) and Luze et al. (2021) suggest that the use of PPE can lead to reduced endurance, impaired cognitive performance and increased risk of accidents [11, 12]. In the context of COVID-19 pandemic, HCW in India and Singapore reported heat stress symptoms like increased thirst, sweating, and exhaustion when wearing PPE [13]. Similarly, a German study observed occupational heat stress among HCW using PPE on hot days. In this questionnaire-based study, involving a total of 428 participants, 96.5% of the participants described their work on hot days as exhausting, 93.0% reported respiratory problems and 85.8% had difficulties concentrating on their tasks [8]. A Dutch study observed that HCW were 25 times more likely to experience heat stress symptoms when performing medical tasks with PPE compared to performing tasks without PPE [14]. Moreover, a recent study has shown that wearing PPE not only leads to heat exhaustion, but also negatively affects mood, motor function and overall task performance [15]. Although HCW may be aware of these adverse health effects, the incorporation of best practices for the mitigation of heat stress into daily routines proves challenging, e.g., due to limited staff capacity, infrastructure or funding. However, it is the employer’s responsibility to protect employees from occupational heat stress, as emphasized by the guidelines from the German Ministry of Labor and Social Affairs [10, 16, 17]. Thus, there is an urgent need for practical and validated concepts for improve occupational safety [18].

In summary, the aforementioned studies collectively highlight the challenge of heat stress for HCW, particularly considering the impact of the changing climate. However, it is important to acknowledge the limitations of these studies and the need for more comprehensive research. Two studies collected retrospective data based on self-report questionnaires to assess heat stress [8, 14]. Other studies lacked the standardization of heat stress exposure due to real-life conditions, potentially obscuring individual effects of heat stress or PPE, such as the ability to self-pace [12, 13]. One study investigated the effects of heat stress and wearing PPE under controlled conditions in a laboratory setting [15]. However, the simplicity of the experimental setup was not comparable to the complex nature of HCW´s real-life tasks.

In response to these gaps, the goal of our study was the investigation of effects on the subjective well-being of HCW by elevated ambient temperatures and PPE using a controlled experimental setting and applying a standardized protocol. We postulate that both temperature as well as PPE exert a negative influence on self-reported well-being.

## Methods

### Study design and population

**Fig. 1:**
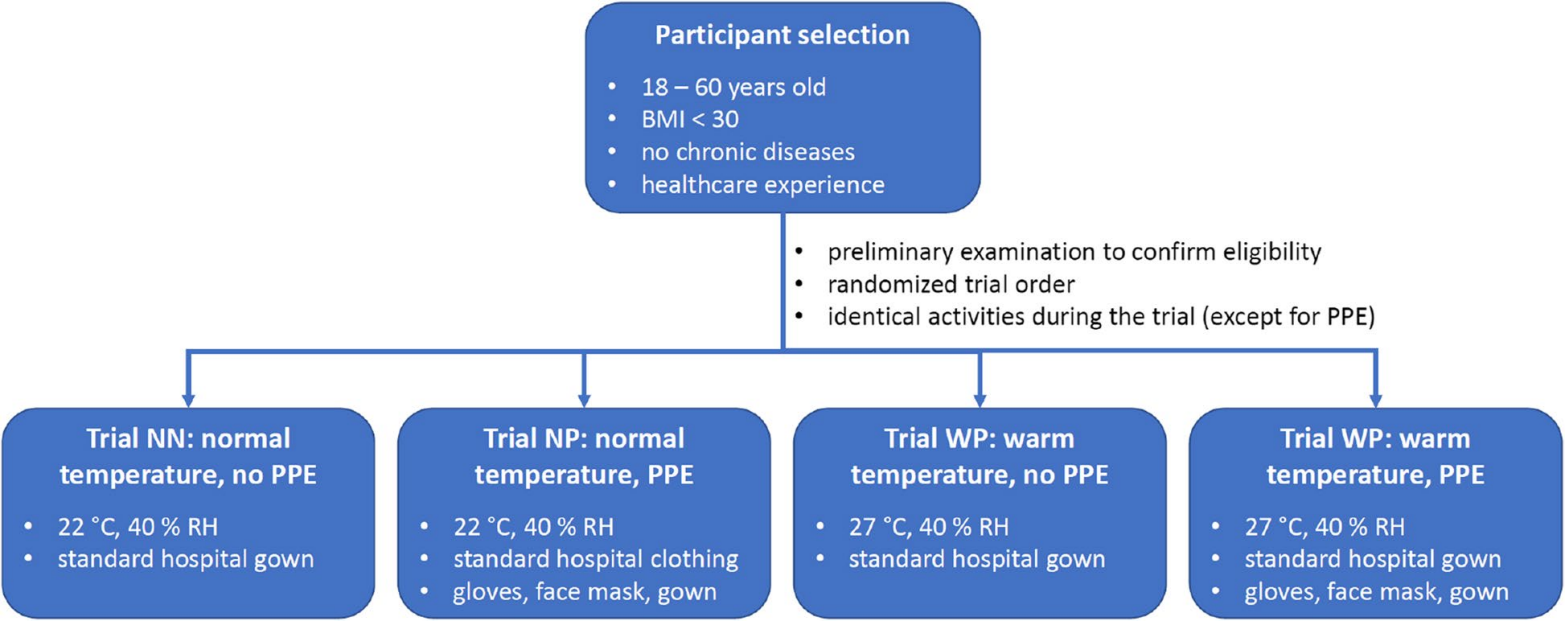
Summary of the experimental procedure

This study was designed as a randomized crossover trial with a total of four independent experiments, which differed in ambient temperature and the use of PPE: 1) normal temperature (22 °C) and no PPE (NN), 2) normal temperature (22 °C) and PPE (NP), 3) warm temperature (27 °C) and no PPE (WN) and 4) warm temperature (27 °C) and PPE (WP) (Fig. 1). The selection of the high temperature was based on the guidelines of the German Federal Ministry of Labour and Social Affairs, where the ambient temperature in workspaces should not exceed 26 °C [17]. However, we chose 27 °C in order to have a sufficient temperature difference to the reference temperature of 22 °C, which was the lower technical limit of the climate chamber. The composition of the PPE was adapted from our hospital regulations for infectious diseases and consisted of protective gloves, FFP2 masks, a disposable plastic gown and a face shield.

Participants were recruited using convenience sampling through both offline and online postings at the LMU Munich University Hospital. Inclusion criteria comprised individuals aged 18 to 60 years with a background or experience in healthcare-related activities. Exclusion criteria included sensitivity to heat (e.g., dizziness, redness on the skin), obesity (BMI > 30) and severe chronic diseases such as COPD or acute coronary heart disease. The study team conducted a preliminary examination to confirm eligibility for participation. All participants provided their informed consent through signed consent forms and were granted a 300 € allowance after completion of the entire study. Data collection was performed between October 2021 and March 2022.

### Climate chamber and experimental procedure

The climate chamber at the University Hospital Munich is a 5 m by 3 m by 2.2 m (L/W/H) room (33 m^3^) connected to an external air-conditioning unit. The chamber was accessed via a windowed safety door that could be opened from both sides. Communication with participants was conducted by either eye contact, hand signs or via an intercom system. In this room, a patient care situation was simulated. In detail, the interior included a table with a chair, a treadmill, a patient bed and a patient dummy (CLA1®, 21 kg, Coburger Lehrmittelanstalt, Coburg, Germany). A picture of the experimental setting can be found in the supplementary information (Figure S1). The temperature in the chamber was controlled and set to either 22 °C or at 27 °C depending on the experiment. The relative humidity was set to 40% for all experiments to limit the number of variables. Climatic conditions in the chamber were monitored using a *QUESTemp 34* Heat Stress Monitor® (TSI, Shoreview, Minnesota, USA), which was placed on a table at a height of 1 m.

The procedure of all trials was identical with the exception of wearing and changing PPE. Participant arrived at the chamber and changed into a standard hospital gown. Thereafter, they entered the chamber to follow a standardized 3.5-h protocol, which can be found in the supplementary information. The protocol was developed under supervision of an experienced HCW at the State Vocational School for Nursing at the University Hospital of LMU Munich. It simulated a typical healthcare setting during a regular shift and included taking care of a patient (mobilization, washing, changing clothes), walking and sitting at a table (administrative tasks). In experiments involving PPE (NP, WP), participants were required to put on and take off PPE at multiples times during the experiment. Participants were allowed to drink water (room temperature) throughout the experiment. The amount of total consumed water was noted. Every participant conducted each experiment at the consistent time of the day (mornings or afternoons). The interval between two experiments was at least one week, but not longer than ten days. The order of experiments was randomized for each participant.

### Questionnaires

To assess participants’ subjective well-being during the experiments, a questionnaire in German language consisting of several survey instruments was used. An English translation of the questionnaire as well as the original document can be found in the supplementary information. Prior to each trial, the participants assessed their perceived personal state of health using a visual analog scale ranging from 0 (worst health status) to 10 (best health status). After each trial, participants were once again asked to assess their perceived personal state of health. Moreover, thermal, physiological and psychological stress were evaluated using the same scale ranging from 0 to 10. Questions about the effect of heat and PPE on personal restrictions and perceived health issues were adapted from a previous HCW survey [8]. Possible responses on the 4-point Likert scale were “no “, “rather no”, “rather yes” and “yes”. Furthermore, the NASA task load index (NASA-TLX, scale ranging from 0 to 10) was included to evaluate the influence the effect of heat and PPE on the perceived workload [19]. Finally, the participants were asked to provide free-text answers if they experienced anything unusual not covered by the questionnaire.

### Data presentation and statistical analysis

Excel (Version 16.0, Microsoft Corporation®, Redmond, USA) was used for initial data processing. The results were stratified by experimental condition as well as by experimental order. Due to the non-parametric distribution of the data, Friedman-Tests with Bonferroni correction (α = 0.05, SPSS®, Version 29.0, IBM, Armonk, USA) were used for outcomes with continuous results. For outcomes using a 4-point Likert scale (“no”, “rather no”, “rather yes” and “yes”), a descriptive analysis was performed based on the share of each answer. The visualization of the results was performed using R Statistical Software® (version 4.0.0). For box-whisker plots, individual differences between the reference experiment NN and the other experiments (NP, WN and WP) were calculated.

## Results

### Participant characteristics and climate chamber conditions

23 eligible participants were evaluated in the preliminary examination. Of those, 18 were finally included in the study. A summary of the participants’ physiological information can be found in Table 1. Eleven participants were females and seven participants were actively working as nurses at the time of the study. The other participants were from different occupational backgrounds in the health sector (medical and health science students, laboratory assistants or hospital administrative staff with nursing background). The average time between the experiments was 8.5 days. For trials at normal temperatures (NN and NP), the average dry bulb temperature was 23.0 ± 0.9 °C (heat index of 21.2 ± 1.2 °C). For trials at warm temperatures (WN and WP), the average dry bulb temperature and the value was 27.3 ± 0.7 °C (heat index of 27.2 ± 0.8 °C). The measured mean relative humidity for all settings was 34 ± 5.2%.

**Table 1:** Participant’s characteristics at the time of the preliminary examination (*n* = 18)

Parameter	Mean ± SD
Age in years	35.2 ± 10.4
Height in m	1.71 ± 0.09
Weight in kg	70.3 ± 15.8
BMI in kg/m2	23.9 ± 4.2
Blood pressure (sys/dia) in mmHg	137/87 ± 19/11
Heart rate in beats/min	73 ± 11

### Questionnaires

**Fig. 2:**
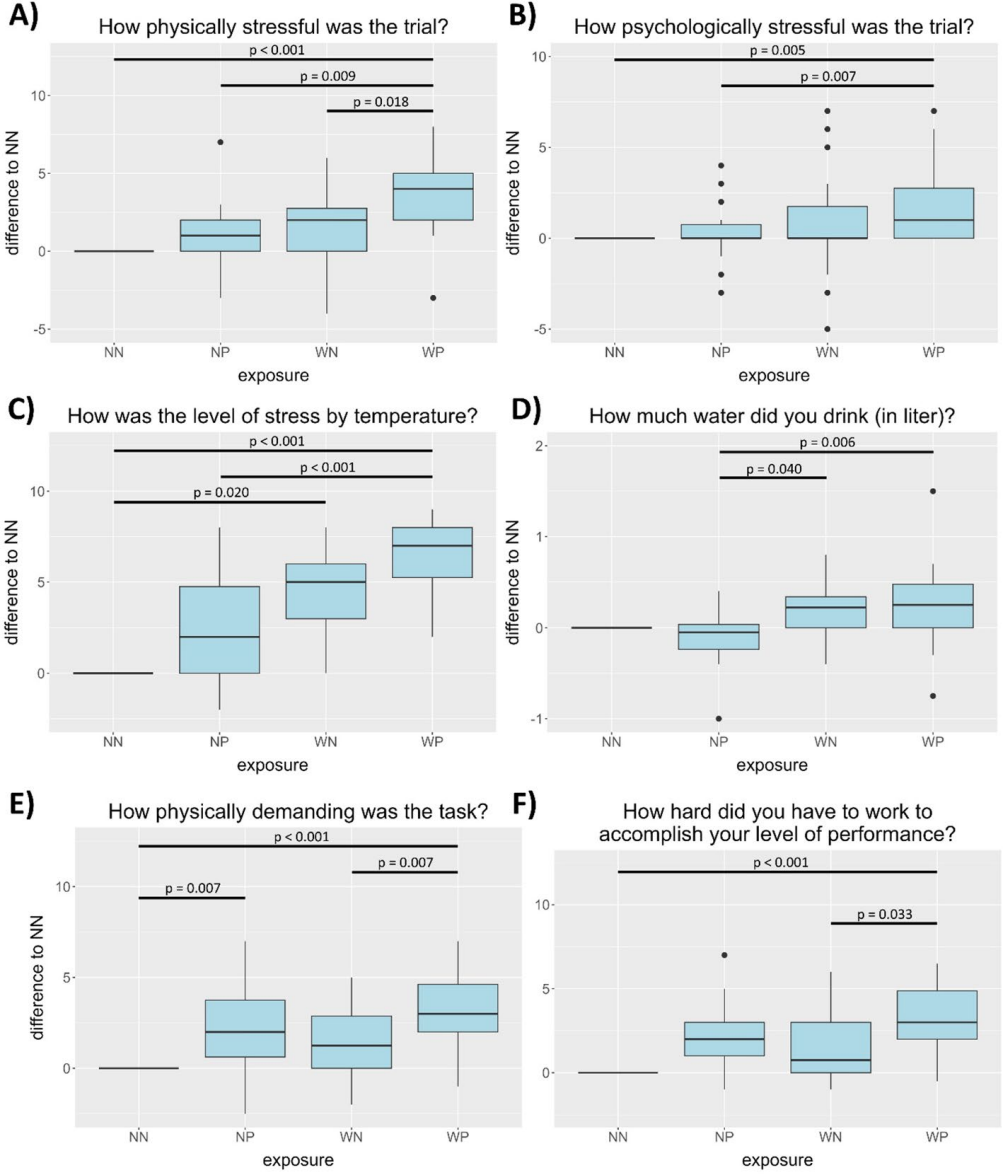
Box-whisker plots for outcomes with statistically significant differences (Friedman rank sum test with Bonferroni correction for multiple testing). The difference in the score for each experimental conditions (NP: 22 °C, PPE; WN: 27 °C, no PPE; WP: 27 °C, PPE) were calculated in relation to the reference condition (NN, 22 °C, no PPE (0))

This study aimed to examine the subjective well-being of HCW when exposed to heat and PPE under controlled conditions using questionnaires. All questionnaires were completely answered by all participants. The results for outcomes with continuous variables and statistically significant differences between the experimental conditions are shown in Fig. 2. The plots display the individual difference of the experimental conditions NP, WN and WP compared to the reference condition NN.

Statistically significant differences were found for physical (A), psychological (B), and thermal stress (C). For physical and thermal stress, higher median scores were observed for temperature and PPE alone as well as for the combination of both factors. Warmer temperatures had a stronger effect on the response than PPE alone. The highest level of physical and thermal stress was reported for the combination of warm temperatures and PPE. For physical stress, the effect was significant for the combination of temperature and PPE compared to all other conditions. For thermal stress, the effect was significant for warmer temperatures compared to normal temperatures and for the combination of temperature and PPE compared to normal conditions and PPE alone. A median increase of the psychological stress score was observed for the combination of temperature and PPE, although less pronounced compared to physical and thermal stress. Furthermore, higher temperatures had a statistically significant effect on water consumption, but not wearing PPE (D). Statistically significant differences were also found for physical demand (E) and effort (F). The median score for physical demand was reported to be higher when wearing PPE compared to being exposed to higher temperatures. This difference was significant for wearing PPE alone compared to normal conditions as well as for wearing PPE at higher temperatures compared to normal conditions and higher temperatures alone. Participants’ median score of effort for accomplishing the experiments were highest when PPE was worn at higher temperatures. However, the increase was only significant for the combination of higher temperature and PPE compared to normal conditions and higher temperatures alone. In contrast, no statistically significant differences were found for overall state of health, mental demand, temporal demand, performance and frustration (Figure S2). Nevertheless, the median temporal demand was higher in experiments with PPE and high temperatures. Similarly, the median perceived success was reduced when PPE and higher temperatures were combined.

**Fig. 3:**
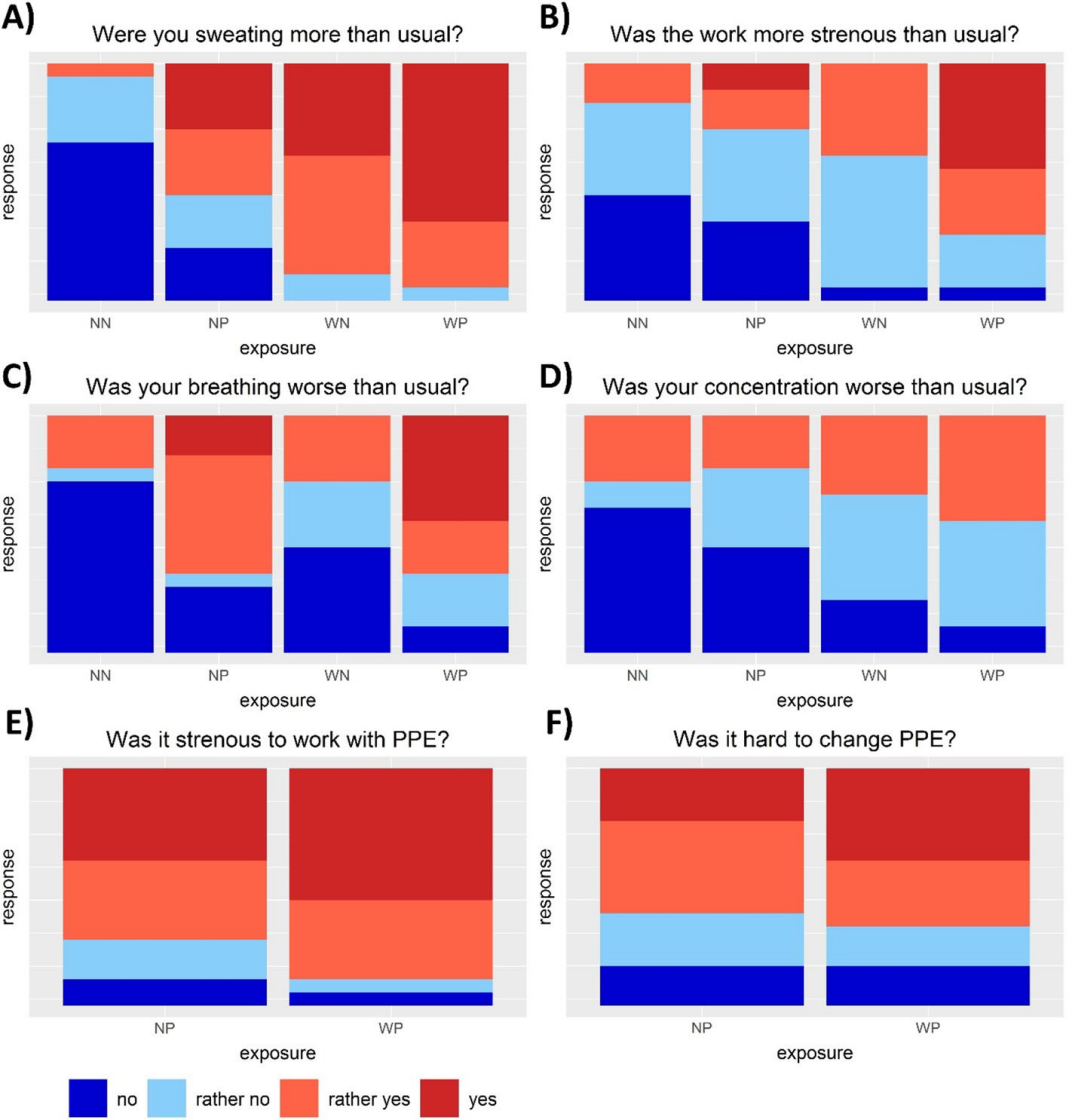
Bar plots of reported physical challenges during the individual experiments (NN: 22 °C, no PPE; NP: 22 °C, PPE; WN: 27 °C, no PPE; WP: 27 °C, PPE). Each color represents the share of the respective answer on a 4-point Likert scale (“no”, “rather no”, “rather yes” and “yes”) in relation to the number of all answers (*n* = 18)

The descriptive results for physical challenges during the experiments (4-point Likert scale) are shown in Fig. 3. A high percentage of participants reported that they were sweating more than usual when working under warm conditions or with PPE (A). When PPE was combined with warm conditions (WP), almost all the participants reported increased sweating. Similarly, participants experienced work under warm temperatures and with PPE as more strenuous, although this observation was less pronounced (B). In contrast, breathing problems were reported more frequently reported for experiments with PPE, which was intensified by the combination with higher temperatures (C). However, warmer temperatures alone had a smaller effect on the response. For concentration, the share of participants that reported no problems declined with PPE and higher temperatures (D). In addition, more participants reported that working with PPE was more strenuous at warm temperatures than at normal conditions (E). Similarly, changing PPE was perceived harder by more participants at higher temperatures (F). No or small differences in the responses were observed when the participants were asked if they were irritable, nervous, disappointed or exhausted (Figure S3).

**Fig. 4:**
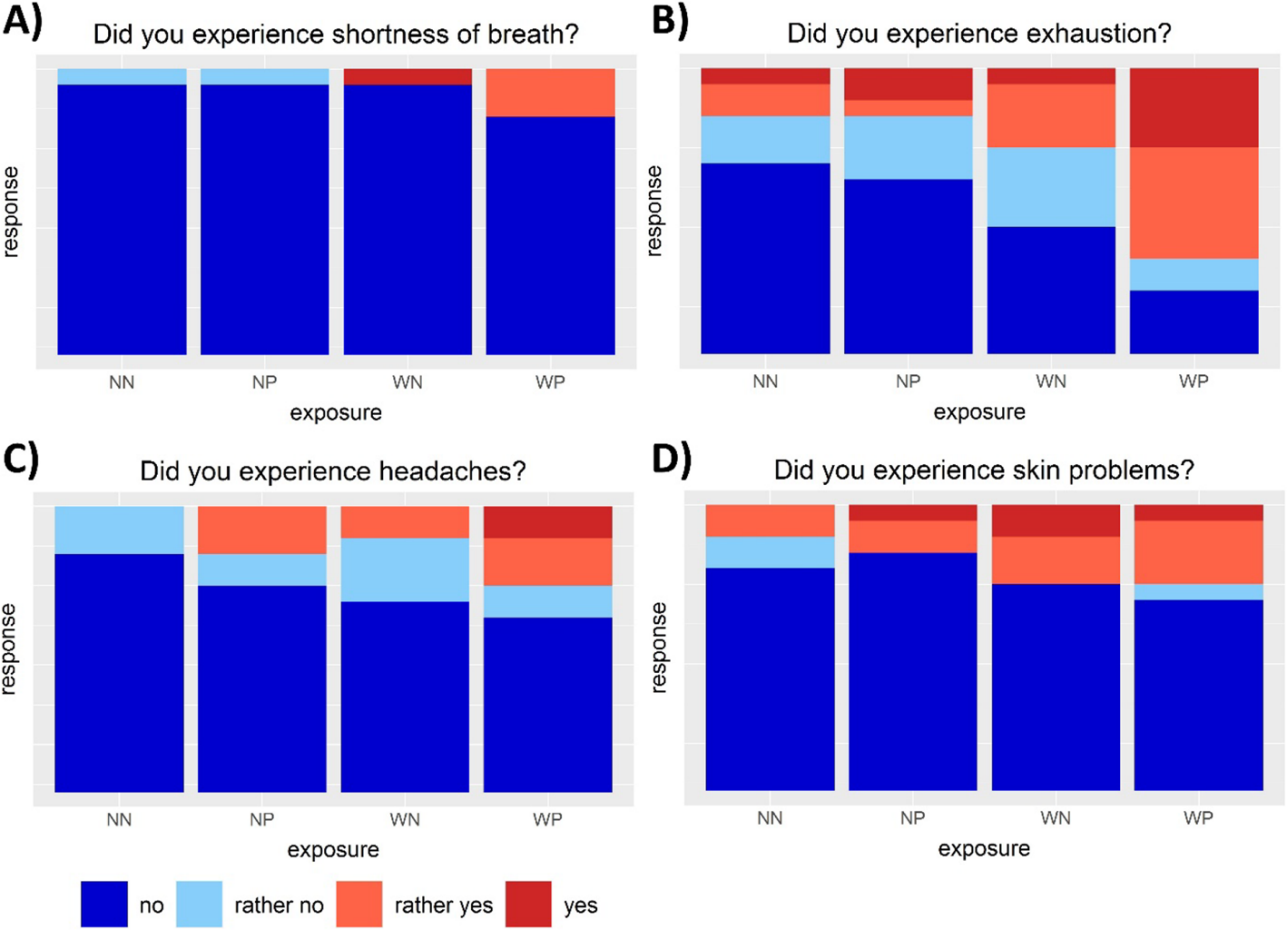
Bar plots (*n* = 18) of experienced health issues during the individual experiments (NN: 22 °C, no PPE; NP: 22 °C, PPE; WN: 27 °C, no PPE; WP: 27 °C, PPE). Each color represents the share of the respective answer in relation to the number of all answers (*n* = 18)

The results for health issues experienced during the experiments are shown in Fig. 4. Almost no participants reported shortness of breath for either wearing PPE or higher temperatures (A). In contrast, slightly more participants reported shortness of breath when higher temperature and PPE were combined. The participants felt most exhausted when they had to work with PPE at higher temperatures (B). Headache (C) and skin problems (D) were experienced more frequently by the participants when using PPE at higher temperatures, although not as much as exhaustion. None to very little differences in the response were observed for intestinal problems, dizziness, fatigue and insecurity (Figure S4).

No statistically significant differences were found when the results were stratified by the sequence of experiments. However, small changes of the median score were found for some parameters (Figure S5). In fact, slightly more participants reported that they were nervous during the first experiment compared to the following experiments. In contrast, headaches were experienced more frequently during the experiments 2 to 4. Furthermore, a lower median score for mental demand for the experiments 2 to 4 in comparison to the first experiment was observed.

Encouraged to share individual experiences not covered by the questionnaire, the participants frequently reported less motivation after the second experiment. Furthermore, boredom was often reported by the participants because they already knew the procedure after two or three trials. Nevertheless, the majority of participants stated that the trials under warm conditions and with PPE were perceived as most uncomfortable.

## Discussion

To the best of our knowledge, this study introduces a novel approach using a crossover exposure design to control both climatic conditions and the healthcare-related tasks performed by the participants to examine the subjective well-being of HCW when exposed to heat and PPE. Given the growing significance of these conditions in the context of climate change and the spread of infectious diseases such as COVID-19, we employed a standardized protocol that simulated a patient care situation in a hospital setting.

Overall, we found that the combination of wearing PPE at higher temperatures resulted in the highest reported perceived discomfort for the majority of outcomes. Elevated ambient temperatures are known to increase core body and skin temperatures, particularly in combination with increased physical activity. This can impact both physical and cognitive performance [3, 4, 7]. It has been demonstrated that PPE can exacerbate these effects, possibly due to reduced ability to dissipate excess body heat [11, 15]. The results of this study are in line with prior publications on HCW exposed to heat while wearing PPE [8, 13, 14, 20]. Therefore, the developed protocol in this study proves effective in replicating the HCW experience within a controlled environment.

As highlighted in a study conducted by Luze et al., PPE demonstrated an impact on thermal stress even at normal ambient temperatures [12]. This aligns with participants reporting increased sweating when wearing PPE at normal temperatures. Within the same project, we have also investigated physiological parameters and found a positive correlation of heat and PPE with body and skin temperature [21]. Thus, the reported effect of heat and PPE on sweating and thermal stress are very likely connected to the increase in body and skin temperature.

Despite participants reporting higher thermal stress and increased sweating when wearing PPE, there was no significant increase in water consumption compared to trials without PPE. In fact, water consumption was only significantly increased at higher temperatures. Unfortunately, our study faced limitations in assessing the quantify of sweat, as there might be a notable difference between reported and actual sweating.

We observed that elevated temperatures, especially in combination with PPE, led to increased exhaustion and a perception of the work being more strenuous. This may be attributed to a diminished capacity for physical recovery, partly due to worse breathing associated with PPE. Consequently, perceived performance, success, effort and physical demands can be adversely affected by the combination of heat and PPE. While this may not directly affect HCW itself, it represents a potential risk for the patients. A decline in HCW performance may consequently lead to a decline in the quality of their work [8]. In the worst case, patients may not receive the appropriate care they need.

However, we did not observe significant effects of heat and PPE on irritation, nervousness, disappointment, insecurity and mental demand. This may be a result of the experimental design of the study, where the participants had sufficient time to complete their tasks. In real-life healthcare settings, the temporal challenge is likely much higher and could affect the performance when exposed to heat.

## Limitations

The sample size calculation for this study was performed for the measurement of physiological parameters [21]. Consequently, the recruitment of more participants could have been beneficial for the analysis of subjective outcomes. Nonetheless, we were able to identify differences between the experimental settings despite the relatively small sample size due to the crossover design of this study.

We recognize that the complexity of the tasks performed in the climate chamber is different compared to real-life settings. Nevertheless, we have tried to tailor the tasks as closely as possible to the real working life of HCWs. Furthermore, we used a factorial to assess individual as well as possible combination effects. Although the mix of different activities was designed to represent the daily routine of an HCW, participants reported that the tasks were too repetitive, that they sometimes felt bored or that the available time to complete each task was too long. Some participants missed the usual background noise or communication with the patient or colleagues. This feedback needs to be taken into account to improve the design of future studies, e.g., by introducing artificial sound effects or varying the tasks.

The temperatures selected in this study reflect the situation in Germany where 22 °C is considered a normal or desirable indoor temperature, whereas 27 °C is considered an undesirable indoor temperature. It is plausible that the impact of higher indoor temperatures, e.g., in tropical regions, could be more severe compared to those used in this study.

## Conclusion

Our study demonstrated that our experimental design utilizing a standardized protocol and questionnaire was suitable to simulate and assess thermal discomfort induced by heat, PPE or the combination of both. From an occupational health point of view, it is crucial to identify the exposure to heat and PPE as an unfavorable working environment for HCW as our results indicate that heat and PPE may result in reduced concentration, exhaustion and impaired tasks performance, which may negatively impact patient care. Although no severe adverse health effects were observed in our trials, real-life exposure to heat may occur over multiple days and at even higher ambient temperatures. Thus, adverse health effects are more likely.

Considering the expected increase of heat waves and elevated temperatures, it is important to protect HCW from heat-related stress. To mitigate potential health effects of heat exposure during working hours, employers should proactively implement protective measures for HCW. Based on this study, the design can be adapted for testing additional factors contributing to thermal discomfort and, more importantly, for the evaluation of the effectiveness of potential interventions such as cooling vests [12].

### Supplementary Information

**Supplementary Material 1.**

## Data Availability

The data can be made available upon reasonable request.
