# Supplementary material for: Assessment of subjective well-being of healthcare workers in response to heat and personal protective equipment under controlled conditions using a standardized protocol
Source: J Occup Med Toxicol. 2024 May 15;19:16. doi: 10.1186/s12995-024-00418-5 (PMC11095016; doi:10.1186/s12995-024-00418-5)

Figure S1: Pictures of the climate chamber setup

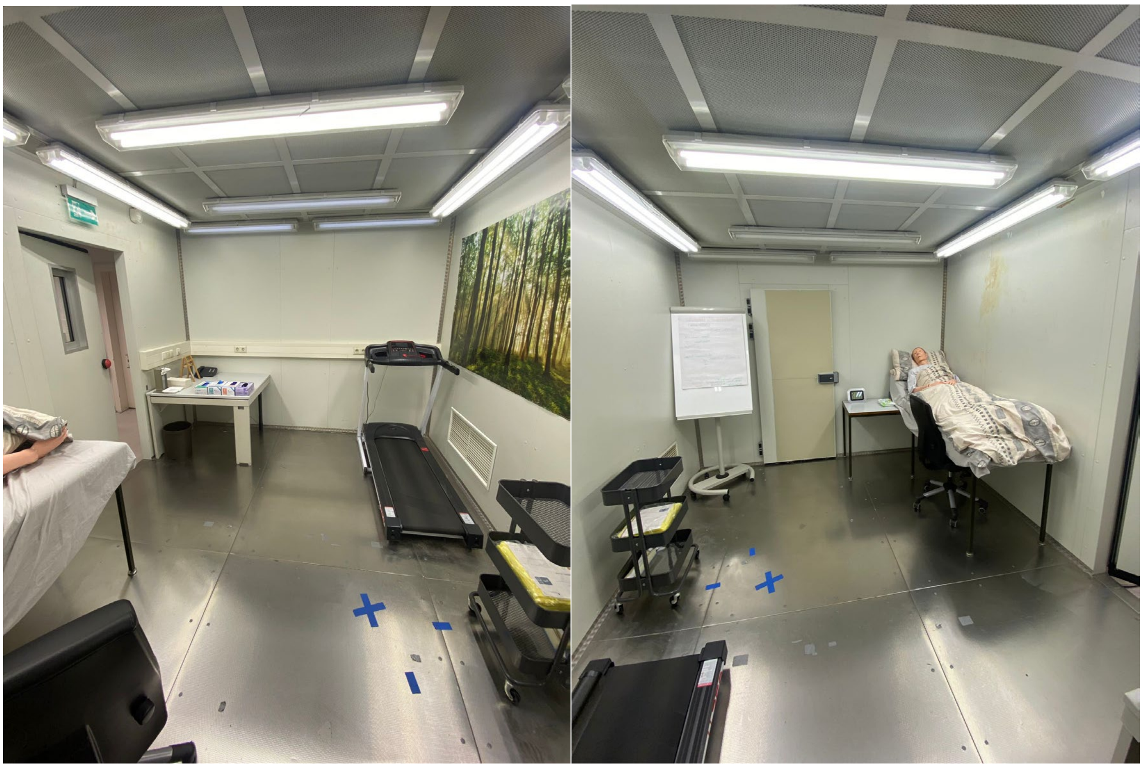

Figure S2: Additional Box-whisker plots of questionnaire responses stratified by experimental condition

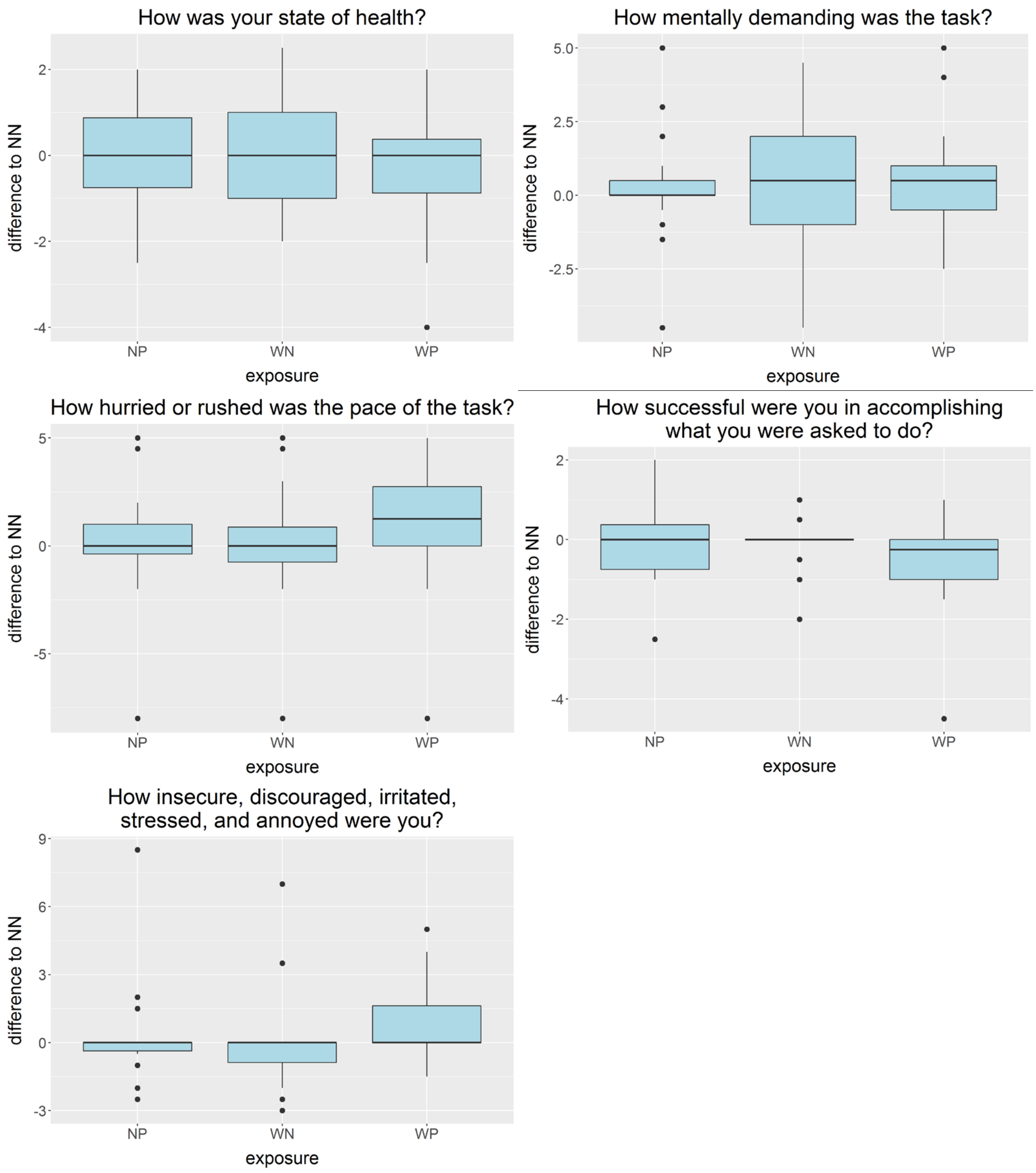

Figure S3: Additional bar plots stratified by experimental condition for experienced physical challenges.

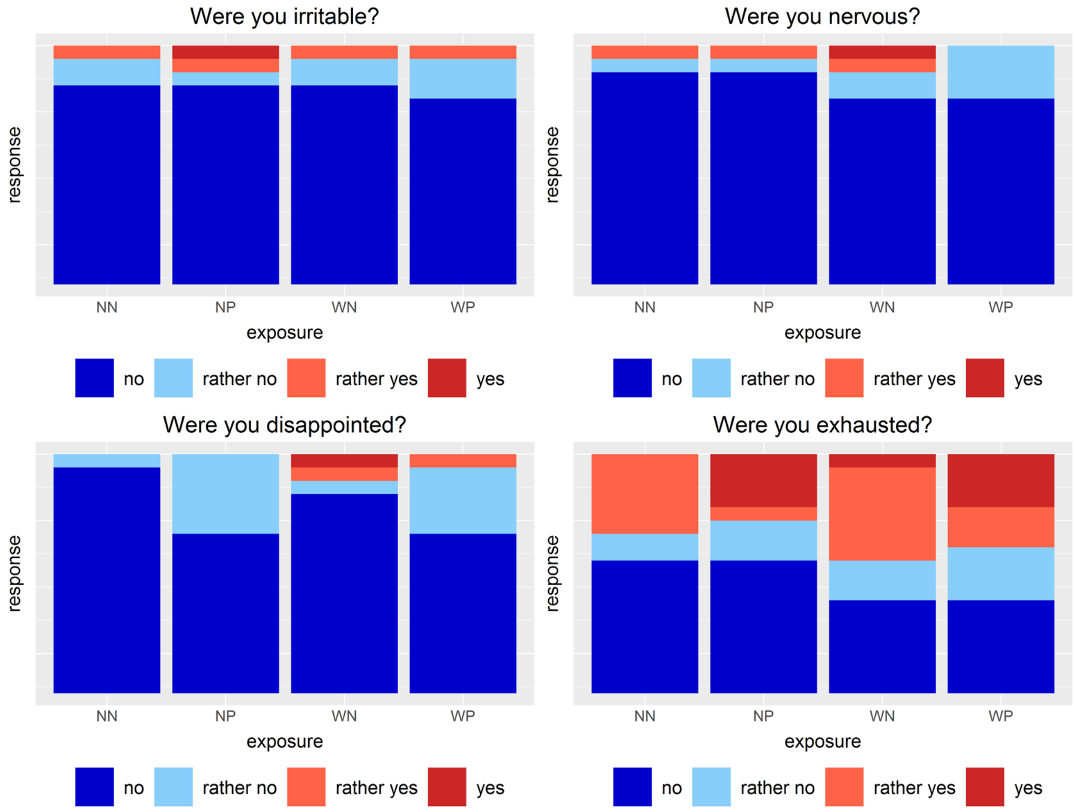

Figure S4: Additional bar plots stratified by experimental condition for experienced health issues.

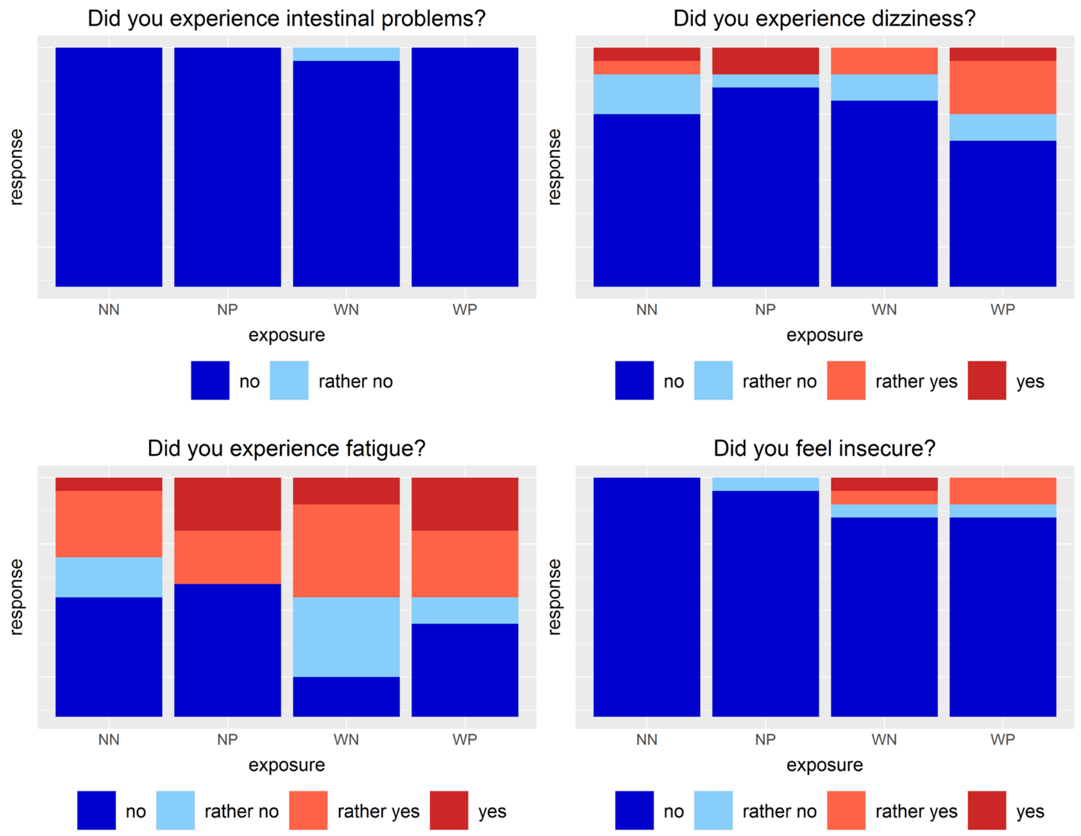

Figure S5: Box-Whisker and Bar Plots stratified by experimental order

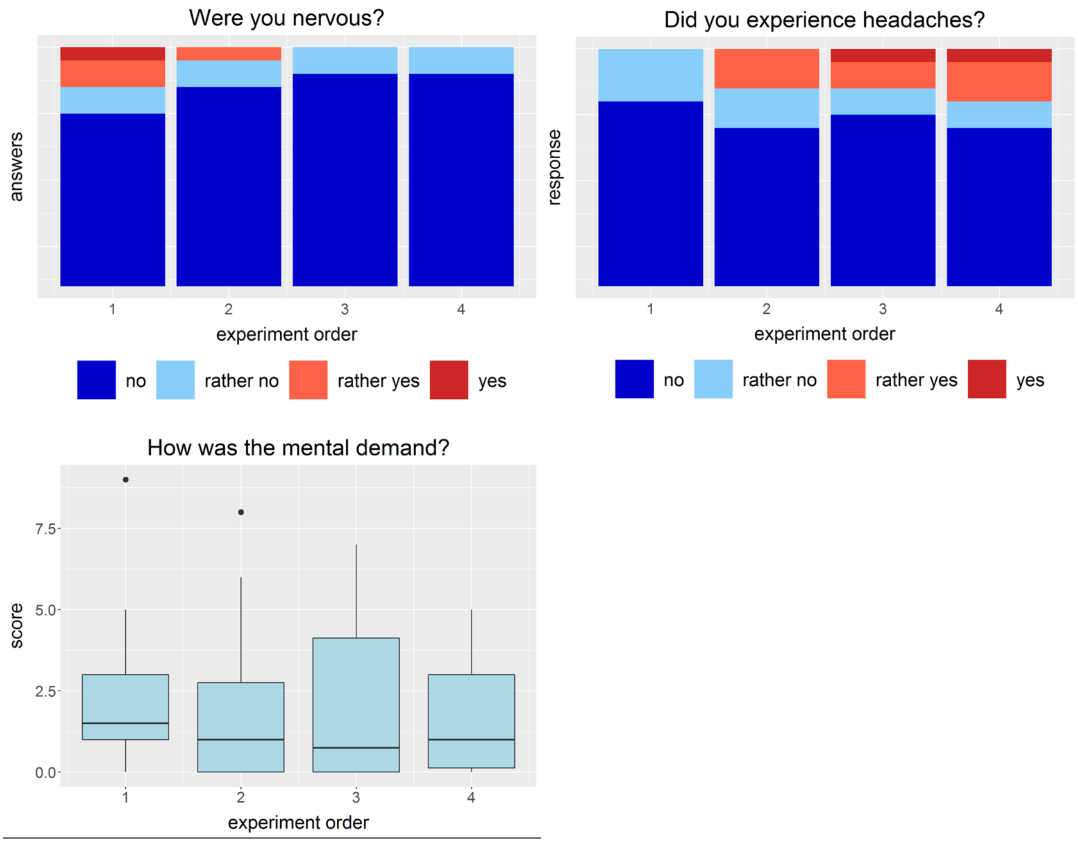

Supplement: Supplementary file 1 — Supplementary Material 1. [file 12995_2024_418_MOESM1_ESM.zip › SI_additional graphs.pdf]
