# Supplementary material for: Assessment of subjective well-being of healthcare workers in response to heat and personal protective equipment under controlled conditions using a standardized protocol
Source: J Occup Med Toxicol. 2024 May 15;19:16. doi: 10.1186/s12995-024-00418-5 (PMC11095016; doi:10.1186/s12995-024-00418-5)
Supplement: Supplementary file 1 — Supplementary Material 1. [file 12995_2024_418_MOESM1_ESM.zip › SI_Questionnaire_English (Translation).pdf]

LMU KLINIKUM
Institut und Poliklinik für Arbeits-, Sozial- und Umweltmedizin

Probanden ID: _______

Questionnaire exposure climate chamber

Date: ___/___/___ [dd/mm/yy]

Examiner: ________________________

Ambient conditions in the climate chamber:

◊ normal (22°C) ◊ warm (27°C)

Experimental conditions:

◊ PPE ◊ no PPE

Participant's information before the experiment:

Height: _____ cm Weight: _____ kg

Blood Pressure: _____/_____/_____ mmHg [Sys/MD/Dia]

Heart rate: _____ bpm

Body temperature: _____°C

Time:

Participant's information after the experiment:

Weight: _____ kg

Blood Pressure: _____/_____/_____ mmHg [Sys/MD/Dia]

Heart rate: _____ bpm

Body temperature: _____°C

Time:

Miscellaneous:

____

____

LMU KLINIKUM
Institut und Poliklinik für Arbeits-, Sozial- und Umweltmedizin

Probanden ID: _______

1. How is your state of health before the experiment?

10 means the best "health" you can imagine. 0 means the worst "health" you can imagine. Mark an X on the scale to indicate how you feel about your health right now.

Health means what you imagine being healthy to be.

Now please write the number you marked on the scale in the box below.

Your current health is _____.

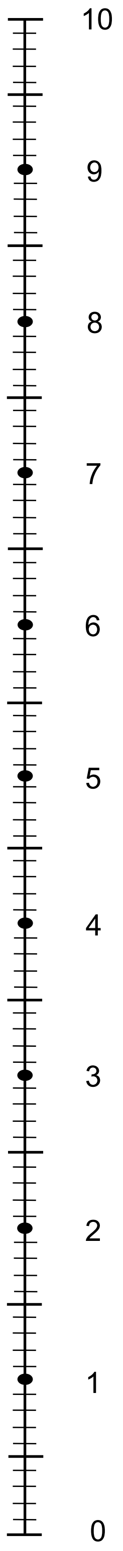

Probanden ID: _______

2. How physically stressful was the trial? (0 = not stressful at all; …., 10 = maximum stressful)? _____

3. How psychologically stressful was the trial? (0 = not stressful at all; …., 10 = maximum stressful)? _____

4. How much water did you drink today? ______ liter

5. How was the level of stress by temperature? (0 = not stressful at all; …., 10 = maximum stressful)? _____

6. Did you experience problems?

6.1. Was the work more strenuous than usual?

◊ yes ◊ rather yes ◊ rather no ◊no

6.2. Were you sweating more than usual?

◊ yes ◊ rather yes ◊ rather no ◊no

6.3. Was your breathing worse than usual?

◊ yes ◊ rather yes ◊ rather no ◊no

6.4. Was your concentration worse than usual?

◊ yes ◊ rather yes ◊ rather no ◊no

6.5. Was it hard to change PPE?

◊ yes ◊ rather yes ◊ rather no ◊no ◊ not applicable (no PPE)

6.6. Was it strenuous to work with PPE?

◊ yes ◊ rather yes ◊ rather no ◊no ◊ not applicable (no PPE)

7. How did you feel today?

7.1. Nervous:	◊ yes	◊ rather yes	◊ rather no	◊no
7.2. Irritable:	◊ yes	◊ rather yes	◊ rather no	◊no
7.3. Exhausted:	◊ yes	◊ rather yes	◊ rather no	◊no
7.4. Dissappointed:	◊ yes	◊ rather yes	◊ rather no	◊no
7.5. Insecure:	◊ yes	◊ rather yes	◊ rather no	◊no

Probanden ID: _______

8. Did you experience any of the following health issues?

8.1. Shortness of breath:	◊ yes	◊ rather yes	◊ rather no	◊no
8.2. Exhaustion:	◊ yes	◊ rather yes	◊ rather no	◊no
8.3. Dizziness:	◊ yes	◊ rather yes	◊ rather no	◊no
8.4. Headaches :	◊ yes	◊ rather yes	◊ rather no	◊no
8.5. Intestinal problems :	◊ yes	◊ rather yes	◊ rather no	◊no
8.6. Skin problems:	◊ yes	◊ rather yes	◊ rather no	◊no
8.7. Fatigue:	◊ yes	◊ rather yes	◊ rather no	◊no

9. Was there anything unusual today (open question)?

____

____

____

LMU KLINIKUM
Institut und Poliklinik für Arbeits-, Sozial- und Umweltmedizin

Probanden ID: _______

10. How is your state of health after the experiment?

10 means the best "health" you can imagine. 0 means the worst "health" you can imagine. Mark an X on the scale to indicate how you feel about your health right now.

Health means what you imagine being healthy to be.

Now please write the number you marked on the scale in the box below.

Your current health is _____.

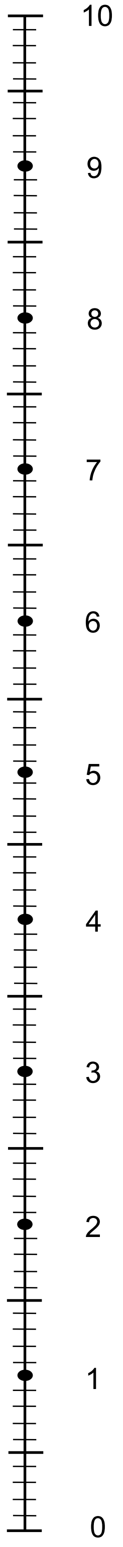

Probanden ID: _______

11. NASA-TLX (Task Load Index)

On the two pages you will find six scales that capture various aspects of stress that contribute to the overall stress of working on a ward.

Please rate your average stress during the past work period on these six scales retrospectively.

Mental Demand

How mentally demanding was the task?

very low — very high

Performance

How successful were you in accomplishing what you were asked to do?

failure — perfect

Physical Demand

How physically demanding was the task?

very low — very high

Effort

How hard did you have to work to accomplish your level of performance?

very low — very high

LMU KLINIKUM
Institut und Poliklinik für Arbeits-, Sozial- und Umweltmedizin

Probanden ID: _______

Temporal demand

How hurried or rushed was the pace of the task?

very low very high

Frustration

How insecure, discouraged, irritated, stressed and annoyed were you?

very low very high
