# Supplementary material for: Assessment of subjective well-being of healthcare workers in response to heat and personal protective equipment under controlled conditions using a standardized protocol
Source: J Occup Med Toxicol. 2024 May 15;19:16. doi: 10.1186/s12995-024-00418-5 (PMC11095016; doi:10.1186/s12995-024-00418-5)
Supplement: Supplementary file 1 — Supplementary Material 1. [file 12995_2024_418_MOESM1_ESM.zip › SI_Questionnaire_German (Original).pdf]

LMU KLINIKUM
Institut und Poliklinik für Arbeits-, Sozial- und Umweltmedizin

Probanden ID: _______

Fragebogen Expositionstag Klimakammer

Datum: ___/___/___ [dd/mm/jj]

Untersucher/in: ________________________

Klimakammerbedingungen

◊ normal (22°C) ◊ heiß (27°C)

Versuchsbedingungen

◊ PPE ◊ ohne PPE

Informationen zum/zur Proband*in zu Beginn des Versuchs:

Größe: _____ cm Gewicht: _____ kg

Blutdruck: _____/_____/_____ mmHg [Sys/MD/Dia]

Puls: _____ bpm

Körpertemperatur: _____°C

Uhrzeit:

Informationen zum/zur Proband*in nach Versuchende:

Gewicht: _____ kg

Blutdruck: _____/_____/_____ mmHg [Sys/MD/Dia]

Puls: _____ bpm

Körpertemperatur: _____°C

Uhrzeit:

Sonstiges:

____

____

LMU KLINIKUM
Institut und Poliklinik für Arbeits-, Sozial- und Umweltmedizin

Probanden ID: _______

1. Wir möchten wissen, wie gut oder schlecht sie sich jetzt, zu Beginn Ihrer Arbeitszeit/ Ihrer Schicht, fühlen.

10 bedeutet die beste “Gesundheit”, die Sie sich vorstellen können. 0 bedeutet die schlechteste “Gesundheit”, die Sie sich vorstellen können. Markieren Sie ein X auf der Skala, um anzuzeigen, wie Sie Ihre Gesundheit im Moment empfinden.

Gesundheit bedeutet, was Sie sich für sich unter Gesundsein vorstellen.

Schreiben Sie nun bitte die Nummer, die Sie auf der Skala markiert haben, in das Feld unten.

Ihre momentane Gesundheit ist _____.

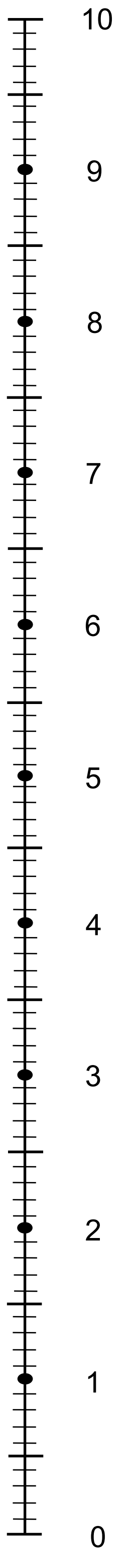

Probanden ID: _______

2. Wie KÖRPERLICH belastend empfanden Sie heute Ihre Arbeit (0= überhaupt nicht belastend; …., 10= maximal belastend)? _____

3. Wie PSYCHISCH belastend empfanden Sie heute Ihre Arbeit (0= überhaupt nicht belastend; …., 10= maximal belastend)? _____

4. Wieviel Flüssigkeit haben Sie während des Versuchs ca. getrunken? ______ Liter

5. Wie empfanden Sie die Temperatur während des Versuchs heute (0= überhaupt nicht belastend; …., 10= maximal belastend)? _____

6. Hatten Sie Probleme während des Versuchs?

6.1. War der Versuch für mich anstrengend:

◊ ja ◊ eher ja ◊ eher nein ◊nein

6.2. Schwitze ich stärker:

◊ ja ◊ eher ja ◊ eher nein ◊nein

6.3. Konnte ich schlechter atmen als üblich:

◊ ja ◊ eher ja ◊ eher nein ◊nein

6.4. Konnte ich mich schlechter konzentrieren als üblich:

◊ ja ◊ eher ja ◊ eher nein ◊nein

6.5. Fand ich das häufige Wechseln von Schutzkleidung besonders anstrengend:

◊ ja ◊ eher ja ◊ eher nein ◊nein ◊nicht zutreffend (ohne PPE)

6.6. War die Arbeit in Schutzkleidung für mich anstrengend:

◊ ja ◊ eher ja ◊ eher nein ◊nein ◊nicht zutreffend (ohne PPE)

7. Wie fühlten Sie sich heute:

7.1. nervös:	◊ ja	◊ eher ja	◊ eher nein	◊nein
7.2. gereizt:	◊ ja	◊ eher ja	◊ eher nein	◊nein
7.3. erschöpft:	◊ ja	◊ eher ja	◊ eher nein	◊nein
7.4. unzufrieden:	◊ ja	◊ eher ja	◊ eher nein	◊nein
7.5. unsicher:	◊ ja	◊ eher ja	◊ eher nein	◊nein

Probanden ID: _______

8. Hatten Sie heute eines oder mehrere der folgenden Probleme?

8.1. Kurzatmigkeit:	◊ ja	◊ eher ja	◊ eher nein	◊nein
8.2. Erschöpfung:	◊ ja	◊ eher ja	◊ eher nein	◊nein
8.3. Schwindel:	◊ ja	◊ eher ja	◊ eher nein	◊nein
8.4. Kopfschmerzen:	◊ ja	◊ eher ja	◊ eher nein	◊nein
8.5. Magen-Darm-Beschwerden:	◊ ja	◊ eher ja	◊ eher nein	◊nein
8.6. Hautprobleme:	◊ ja	◊ eher ja	◊ eher nein	◊nein
8.7. Müdigkeit:	◊ ja	◊ eher ja	◊ eher nein	◊nein

9. Was war heute besonders (offene Frage)?

____

____

____

LMU KLINIKUM
Institut und Poliklinik für Arbeits-, Sozial- und Umweltmedizin

Probanden ID: _______

10. Wir möchten wissen, wie gut oder schlecht sie sich jetzt fühlen.

10 bedeutet die beste “Gesundheit”, die Sie sich vorstellen können. 0 bedeutet die schlechteste “Gesundheit”, die Sie sich vorstellen können. Markieren Sie ein X auf der Skala, um anzuzeigen, wie Sie Ihre Gesundheit im Moment empfinden.

Gesundheit bedeutet, was Sie sich für sich unter Gesundsein vorstellen.

Schreiben Sie nun bitte die Nummer, die Sie auf der Skala markiert haben, in das Feld unten.

Ihre momentane Gesundheit ist _____.

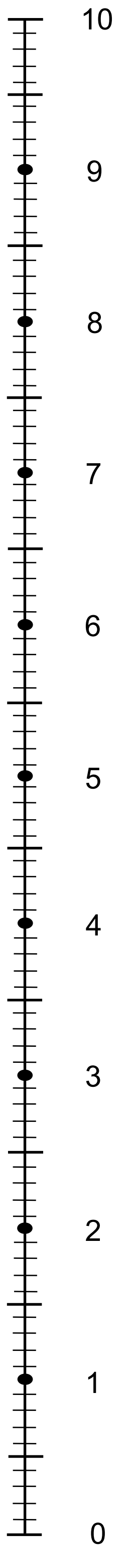

Probanden ID: _______

11. NASA-TLX (Task Load Index)

Auf den beiden Seiten finden Sie sechs Skalen, die verschiedene Beanspruchungsaspekte erfassen, die zu der Gesamtbeanspruchung bei der Arbeit auf Station beitragen.

Bitte schätzen Sie Ihre durchschnittliche Beanspruchung während des zurückliegenden Arbeitsabschnitts auf diesen sechs Skalen retrospektiv ein.

Geistige Anforderungen

Wie hoch waren die geistigen Anforderungen der Aufgabe?

sehr niedrig — sehr hoch

Leistung

Wie erfolgreich haben Sie die geforderte Aufgabe Ihrer Ansicht nach durchgeführt?

Misserfolg — Perfekter Erfolg

Körperliche Anforderungen

Wie hoch waren die körperlichen Anforderungen der Aufgabe?

sehr niedrig — sehr hoch

Anstrengung

Wie sehr mussten Sie sich anstrengen, um Ihre Leistung zu erreichen?

sehr wenig — sehr stark

LMU KLINIKUM
Institut und Poliklinik für Arbeits-, Sozial- und Umweltmedizin

Probanden ID: _______

Zeitliche Anforderungen

Wie hoch waren die zeitlichen Anforderungen der Aufgabe?

sehr niedrig | sehr hoch

Frustration

Wie verunsichert, entmutigt, gereizt und verärgert waren Sie?

sehr wenig | sehr stark
